# Maternal position during the first stage of labor: a systematic review

**DOI:** 10.1186/1742-4755-3-10

**Published:** 2006-11-30

**Authors:** João P Souza, Maria A Miquelutti, Jose G Cecatti, Maria Y Makuch

**Affiliations:** 1 Department of Obstetrics and Gynecology, School of Medical Sciences, Universidade Estadual de Campinas, Campinas, SP, Brazil; 2 CEMICAMP – Center for Studies in Reproductive Health of Campinas, Campinas, SP, Brazil

## Abstract

**Background:**

Policy makers and health professionals are progressively using evidence-based rationale to guide their decisions. There has long been controversy regarding which maternal position is more appropriate during the first stage of labor. This problem has been examined often and repeatedly and the optimal recommendation remains unclear.

**Methods:**

This is a systematic review of the effect of maternal position during the first stage of labor. The main question addressed here is: Does encouraging women to adopt an upright position or to ambulate during the first stage of labor reduce the duration of this stage? All randomized controlled trials carried out to assess this effect were taken into consideration in this review. The following electronic databases were accessed to identify studies: MEDLINE, Popline, the Scientific Electronic Library On-line and the Latin American and Caribbean Health Science Information. Citation eligibility was independently assessed by two reviewers. The methodological quality of each trial was also evaluated independently by two reviewers and a trial under consideration was included only when consensus had been attained. Allocation concealment and screening for the occurrence of attrition, performance and detection biases were considered when studies were appraised. The decision whether to perform data pooling was based on the clinical similarity of studies.

**Results:**

The search strategy resulted in 260 citations, of which 18 were assessed in full-text. Nine eligible randomized controlled trials were included in the systematic review. Randomization methods were not fully described in eight studies. The allocation concealment was considered adequate in four studies and unclear in five. The investigators pooled the data from seven studies in which the length of the first stage of labor and results were in favor of the intervention, but the high level of heterogeneity (I^2 ^= 88.4%) impaired the meaning of this finding. The intervention did not affect other outcomes studied (mode of delivery, use of analgesia, labor augmentation and condition of the child at birth).

**Conclusion:**

Adoption of the upright position or ambulation during first stage of labor may be safe, but considering the available evidence and its consistency, it cannot be recommended as an effective intervention to reduce duration of the first stage of labor.

## Background

Even before the development of modern obstetrics, controversy existed with respect to maternal position during labor. The horizontal position during the first stage of labor is believed to have been introduced by Mauriceau in the 18^th ^century to facilitate the care of women and the performance of obstetric maneuvers and procedures [1]. The horizontal position was thus incorporated into Western culture as the standard position during labor. Nevertheless, the standardization of this position for labor was never fully accepted, and more than two centuries ago there were those who advocated the value of not confining women in labor to bed [2]. In fact, in most cultures that have not been influenced by this Western custom, women in labor continue to opt for the upright position or to keep ambulating [1].

Throughout the scientific development of obstetrics, this controversy has been examined several times under different perspectives. From the physiological standpoint, the supine position has been observed to be associated with the compression of abdominal blood vessels and impairment of fetal nutrition and oxygenation [3]. It has also been argued that this position would negatively interfere with uterine contractions [4]. However, upright position during first stage of labor may improve maternal comfort and reduce the need for analgesia [5]. In this context, labor without bed confinement became part of a set of actions involved in promoting the empowerment of women and the humanization of labor. In accordance with these views, an argument was built in favor of the upright position during labor.

On the other hand, over the past twenty years, policy makers, health professionals and even the lay society are progressively using an evidence-based rationale to guide their decisions. A considerable amount of knowledge had already been accumulated on the subject more than twenty years ago, and the remaining facts available today have been acquired over that interval of time. In summary, the purpose of the adoption of an upright position has been the enhancement of uterine contractions and fetal condition, and the promotion of maternal comfort [3-5]. Nevertheless, although the issue has frequently been examined, the optimal alternative remains unclear. For this reason, it was decided to carry out a systematic review with the objective of assessing the effect of adopting the upright position or ambulating during the first stage of labor on selected obstetrical and perinatal outcomes.

## Methods

This is a systematic review on the effect of maternal position during the first stage of labor. The main question addressed here is: Does encouraging women to adopt an upright position or to ambulate during the first stage of labor reduce the duration of this stage? All randomized controlled trials comparing upright position or ambulation with any other position were taken into consideration. We defined upright position as the adoption of a not supine position during labor, (i.e. walking, sitting, standing, kneeling, and squatting). The following endpoints were evaluated: need for labor augmentation, mode of delivery, use of analgesia, neonatal condition at birth and maternal comfort and satisfaction.

Studies were identified by performing a search of the following electronic databases: MEDLINE, Popline, the Scientific Electronic Library On-line (SciELO) and the Latin American and Caribbean Health Science Information (LILACS). These databases were searched using the following strategy and keywords: ("labor") AND ("first stage" OR "position" OR "mobility" OR "up right" OR "upright" OR "active phase" OR "latent phase" OR "maximum slope" OR "recumbent" OR "lateral" OR "sitting" OR "standing" OR "ambulation" OR "kneeling" OR "squatting"). This search was not restricted by date or language. In addition, the proceedings of several scientific meetings were hand-checked and reference lists of retrieved publications were screened.

Possible eligibility was assessed independently by two reviewers. Initially, the citations identified were evaluated on the basis of their titles and/or abstracts and full text was retrieved if found to be eligible. All citations considered to be clearly irrelevant were excluded. If the information provided by titles or abstracts was considered insufficient to decide on inclusion or exclusion of the publication, the full-text article was retrieved and evaluated. Studies were assessed independently by two reviewers and decision upon inclusion was based on consensus between the two. Methodological quality assessment took into account the adequacy of allocation concealment. The concealment of allocation was considered adequate (and scored as 'A') when the study under consideration adopted a process in which the person deciding about the inclusion of a participant into a randomized controlled trial did not know the comparison group into which that individual was being allocated. In case allocation concealment was unclear, the score was 'B' (table 1). The occurrence of attrition, performance and detection bias was screened and the sampling method and the mode of presentation of results were also evaluated.

**Table 1:** Main characteristics of included studies

**Study**	**Methods**	**Participants**	**Interventions**	**Outcome**	**Notes**	**Allocation Concealment**
FLYNN [2] (UK, 1978)	Random Generation: not stated Allocation Concealment Method: not stated ("Participants were. randomly allocated")	68 women who expressed an interest in ambulation during labor.	Ambulant group: the intention was to keep the woman ambulant. Recumbent group: the intention was to keep the woman in a recumbent position.	Length of first stage of labor; Labor augmentation; Mode of delivery; and Apgar Score at 5^th ^minute Others (time spent ambulant, contraction frequency and contraction amplitude, basal uterine tone, dose of intravenous or epidural analgesic, Apgar score at 1^st ^minute)	There was no sampling calculation.	B
McMANUS [8] (UK, 1978)	Random Generation: not stated Allocation Concealment by envelopes	40 women with 38 weeks' gestation or more, singleton, with cervical score (Calder, 1974) greater than 5, cephalic presentation and induced labor.	Upright group: the women were encouraged to be up and about. Recumbent group: The women were nursed in the lateral position.	Labor augmentation; Mode of delivery; Analgesia; and Apgar score at 5th minute Others (Induction delivery interval, number of PGE2 tablets, dose of analgesic, Apgar score at 1^st ^minute, Apgar score less than 4, number of women with fetal distress)	There was no sampling calculation. Induced labor	A
READ [9] (USA, 1980)	Random Generation: not stated Allocation Concealment Method: not stated ("patients were prospectively randomized")	14 women in active labor who demonstrated failure to progress over one or more hours, and whose contractions would require augmentation.	Ambulatory group: after the diagnosis of protracted labor, women of this group underwent a 2 h period of walking or standing in an upright position.	Mode of delivery; Apgar score at 5th minute. Others (labor progress characteristics (dilation and station, Apgar score at 1^st ^minute)	There was no sampling calculation. All patients had ruptured membranes. Protracted labor	B
HEMMINKI [10] (Finland, 1983)	Random Generation: not stated Allocation Concealment by sealed envelopes.	627 low risk women who had spontaneous onset of labor, with intact membranes and who were sent from the reception ward to the delivery room during the study period.	Ambulant group: the women were asked by the midwife to be upright or ambulant, but with no obligation and being allowed to rest in the bed whenever they wanted. Control group: the women received the hospital's standard treatment, which means that after arriving in the delivery room they lay in bed, usually on their sides.	Length of labor (first and second stage); Labor augmentation; Mode of delivery; Apgar score at 5th minute; and Analgesia Others (episiotomy, well-being of the fetus, shoulder dystocia, Apgar score at 1^st ^minute, Apgar score less than 7 at 5^th ^minute, days in the hospital, admission to a special care unit, stillbirth and neonatal death)	There was no sampling calculation. Amniotomy was delayed in the study group (co-intervention).	A
HEMMINKI [11] (Finland, 1985)	Random Generation: not stated Allocation Concealment was conducted separately for primipara and multipara by sealed envelopes.	57 women with protracted labor.	Ambulant group: women were encouraged to be upright or ambulant. Oxytocin group: women received the standard treatment provided by the hospital.	Length of labor; Mode of delivery; Apgar score at 5th minute; Women's experiences (maternal comfort). Others (episiotomy, strength of contractions before pushing)	There was no sampling calculation. Protracted labor	A
ANDREWS [12] (USA, 1990)	Random Generation: not stated Allocation Concealment Method: not stated ("Participants were randomly assigned")	40 women "All participants were nulliparous, experiencing a medically uncomplicated pregnancy, with a single vertex fetus in anterior position, spontaneous onset of labor at 38 to 42 weeks' gestation, adequate pelvis measurement, and intact amniotic membranes at the beginning of the phase of maximum slope" (from 4 to 9 cm of dilatation).	Upright position group: the intention was to keep the woman in an upright position. Supine position group: the intention was to keep the woman in a supine position. The women were free to choose several variations within each position group.	Length of the phase of maximum slope during the first stage of labor (4 to 9 cm of dilation); Maternal Confort Score; Analgesic dose; Apgar scores at 1 and 5 minutes.	No sampling calculation. The information on the variability of Apgar scores is not presented (The SD was assumed to be approximately one quarter of the range of the values presented).	B
ALLAHBADIA [13] (India, 1992)	Random Generation: not stated Allocation Concealment Method: not stated ("All patients were selected at random")	200 women with 37 weeks' gestation or more, with adequate pelvis, vertex presentation and no medical, surgical or obstetric disease.	Ambulatory Group: women were 'kept' ambulatory during the first stage of labor and encouraged to adopt the squatting position during the second stage.	Length of labor (first and second stage); Mode of delivery; Incidence of complications (prolonged first stage, prolonged second stage, maternal injuries, perinatal mortality and morbidity)	No sampling calculation. No mention about the beginning of labor (if it was spontaneous or induced). The information on the variability of labor is scarce.	B
BLOOM [14] (USA, 1998)	Random Generation: not stated Allocation Concealment Method: not stated ("The women enrolled in the study were randomly assigned")	1067 women in spontaneous labour with uncomplicated pregnancies between 36 and 41 weeks' gestation, having regular uterine contractions with cervical dilatation of 3 to 5 cm and fetuses in the cephalic presentation.	Walking Group: women were encouraged to walk but were instructed to return to their beds when they needed intravenous or epidural analgesia or when the second stage of labor began. Usual Care Group: women were permitted to assume their choice of supine, lateral or sitting position during labor.	Length of labor (first and second stage); Labor augmentation; Mode of delivery; Analgesia. Others (episiotomy, shoulder dystocia, dose of analgesic, neonatal condition at birth (Apgar score less than 4 at 5th minute, umbilical artery pH<7.0, intubation in delivery room), stillbirth and neonatal death)	No sampling calculation. Women in the control group could assume sitting positions during labor. (Contamination).	B
MIQUELUTTI [15] (Brazil, 2006)	Random Generation: computer generated random sequence (Excel 2003) Allocation Concealment by sealed and opaque envelopes.	107 nulliparous women with uncomplicated singleton pregnancies between 37 and 41 weeks, cephalic, with cervical dilation between 3 to 5 cm.	Study group: the women were encouraged to remain in vertical positions. Control group: the women received usual maternity care.	Length of labor (first and second stage); Mode of delivery; Apgar score at 5th minute; Analgesia; Maternal satisfaction. Others (episiotomy, pain, well-being of the fetus, Apgar score at 1^st ^minute, Apgar score less than 7 at 5^th ^minute)	The sampling calculation was performed. Women in the control group could remain upright if they preferred (Contamination).	A

*Allocation concealment: A = adequate; B = unclear

Data extraction and statistical analysis were performed according to the guidelines recommended in the *Cochrane Handbook *[6]. The decision whether to perform data pooling was based on the clinical similarity of studies. Whenever an alternative measure of variability was applied, an approximation or a direct algebraic relationship was used to obtain the standard deviation. After data pooling, statistical heterogeneity was identified and evaluated as moderate or high (i.e. more than 40% using the I^2 ^statistic) [7]. If heterogeneity was low, a fixed effects model was used for statistical analysis, and if heterogeneity was moderate or high the random effects model was applied. Depending on the heterogeneity level, the standardized mean difference (SMD, low heterogeneity) or the weighted mean difference (WMD, moderate to high heterogeneity) was used. Odds ratios (OR) with 95% confidence intervals (CI's) were calculated. Subgroup analyses according to parity and other characteristics of labor (maximum slope phase and protracted labor) were performed. Analysis was carried out using the Revman software package, version 4.2.8 (The Nordic Cochrane Centre, Rigshospitalet 2003).

## Results

**Figure 1:**
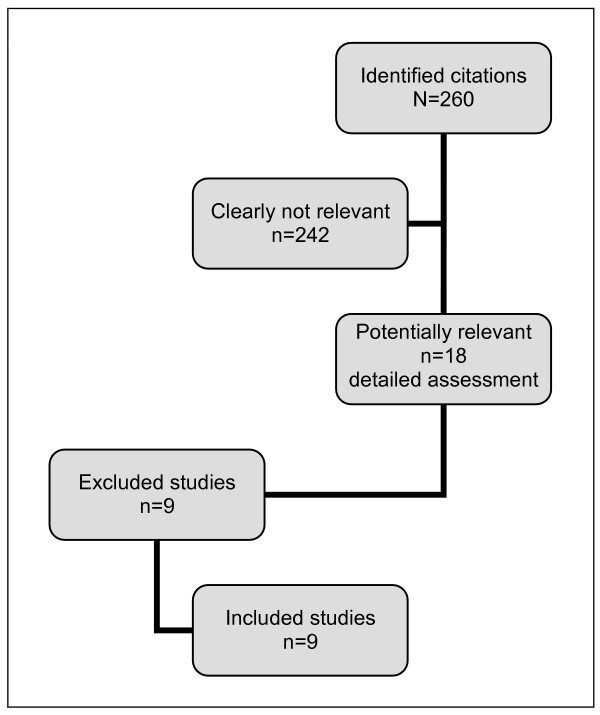
Study selection process.

The search strategy yielded 260 citations, of which 18 were assessed in full-text. Of these, 9 randomized controlled trials were included in the systematic review, with a total of 2,220 women [2,8-15] (Figure 1). None of the studies excluded from the review were randomized controlled trials [1,16-22] and one referred only to the second stage of labor [23]. Main characteristics of studies included in the review are presented in Table 1. The earliest studies were published in 1978 [2,8] and only two were performed in the last ten years [14,15]. All studies were published in English language and two were carried out in developing countries (India [13] and Brazil [15]). Randomization methods were not fully described in eight studies [2,8-14]. The allocation concealment was considered adequate in four studies [8,10,11,15] and unclear in five others [2,9,12-14]. Only one study described sample size calculation [15]. We did not consider blinding to be feasible and, in fact, blinding was not reported in any study included in this review. After data pooling, the heterogeneity was high in three selected comparisons or outcomes. The results mentioned below and the heterogeneity levels are summarized in Table 2.

**Table 2:** Summary of pooled data for all studies included according to selected comparisons or outcomes

**Outcome ^**#**^**	**Studies**	**Participants**	**Statistical Method**	**Effect estimate (95% CI)**	**I^**2**^**
Duration of first stage of labor [2, 10-15]	7	2166	WMD (random)	-0.83 (-1.60, -0,06)	88.4%
Cesarean Section [2, 8-11, 13-15]	8	2180	OR (fixed)	0.98 (0.67, 1.43)	12.0%
Use of analgesia during labor [2, 8, 10, 14, 15]	6	1966	OR (random)	0.69 (0.37, 1.30)	71.8%
Labor augmentation [2, 8, 10, 14]	4	1802	OR (fixed)	0.81 (0.65, 1.01)	0%
Apgar score at the fifth minute [2, 8-11, 15]	6	913	WMD (random)	0.11 (-0.07, 0.28)	67.6%

^# ^studies included

### Duration of first stage of labor

**Figure 2:**
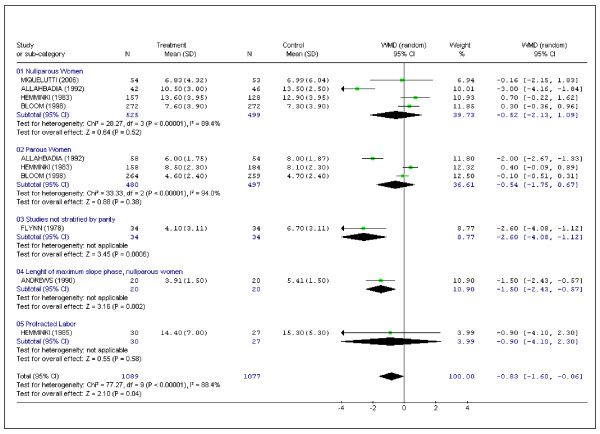
Pooled data for randomized controlled trials in which data were recorded for the duration of first stage of labor (h).

We pooled the data from seven studies in which the duration of first stage of labor was recorded. A total of 2,166 patients were enrolled in those trials [2,10-15]. Two studies accounted for 78.2% (1,694 patients) of all patients enrolled [10,14]. The reviewers observed performance bias in one of these two studies (reporting on 627 randomized women) [10] because of delayed amniotomy in the study group. Another study included in this section randomized only patients with protracted labor [11]. Despite these observations, results favored intervention (WMD (random) -0.83 hours; 95%CI -1.60 to -0.06) (Figure 2).

### Mode of delivery

**Figure 3:**
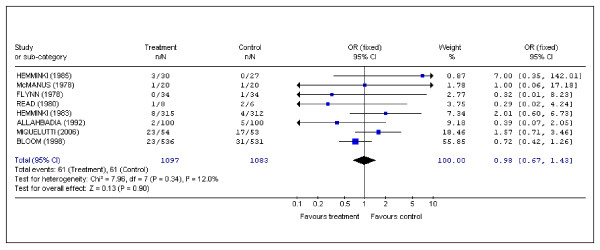
Pooled data for randomized controlled trials in which data were recorded for the occurrence of Cesarean section.

Eight studies examined the mode of delivery [2,8-11,13-15]. The overall cesarean section rate was 5.5% for the intervention group and 5.6% for the control group. (OR (fixed) 0.98; 95% CI 0.67 to 1.43) (Figure 3).

### Use of analgesia

**Figure 4:**
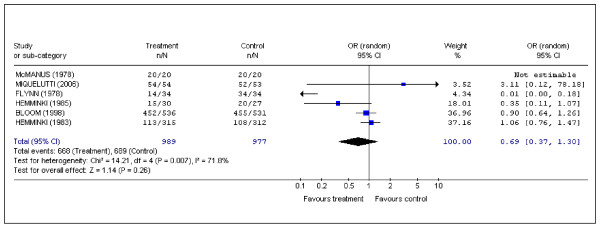
Pooled data for randomized controlled trials in which data were recorded for the use of analgesia.

The use of analgesia was assessed in six studies [2,8,10,11,14,15]. A statistically significant result in favor of the treatment group was found in only one study [2]. The overall use of analgesia was high (69.0%). (Pooled data: OR (random) 0.69; 95%CI 0.37 to 1.30) (Figure 4).

### Maternal comfort

Three studies examined maternal comfort using different methods [11,12,15]. We judged that data pooling would be inappropriate in this case, as the conversion to a common scale was not feasible. In one study [12], a maternal comfort score developed by the authors themselves was used to evaluate maternal reactions to uterine contractions, including certain behavioral and physiological signs. The overall mean comfort score during the first stage of labor did not differ significantly between the two groups. Another study evaluated women's experiences and the results suggest more women rating their experiences positively in the study group compared to the control group [10]. The third study used a visual-analog scale to evaluate maternal satisfaction during labor [15], resulting in no statistically significant difference between the two groups.

### Labor augmentation

**Figure 5:**
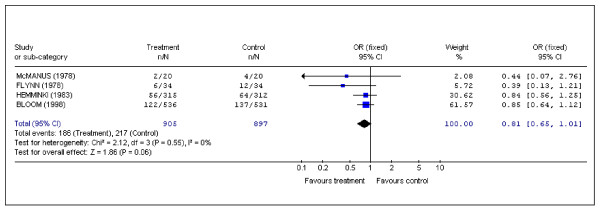
Pooled data for randomized controlled trials in which data were recorded for labor augmentation.

Four studies examined the need for labor augmentation [2,8,10,14]. No statistically significant results were observed, but all of them reported what could be a protective effect. (Pooled data: OR (fixed) 0.81; 95%CI 0.65 to 1.01) (Figure 5).

### Child condition after birth

**Figure 6:**
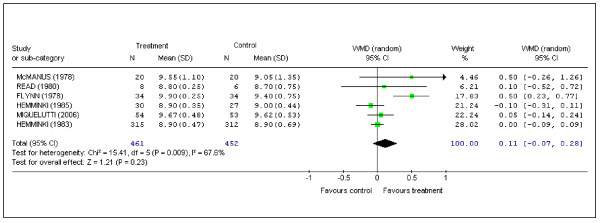
Pooled data for randomized controlled trials in which data were recorded for the Apgar score at the 5th minute of life.

Six studies reported 5-minutes' Apgar scores [2,8-11,15]. One study reported a statistically significant difference in favor of the intervention group [2]. (Pooled data: WMD (random) 0.11; 95% CI -0.07 to 0.28) (Figure 6).

## Discussion

The main result of this systematic review suggests that encouraging women to adopt an upright position or to ambulate during the first stage of labor reduces its duration. However, the robustness of this finding is limited, since it is associated with a high level of heterogeneity (assessed by the I^2^). In fact, the consistency of a meta-analysis depends on the similarity of magnitude of the effects of the studies included, and the assessment of the consistency of effects across studies can be carried out by measuring heterogeneity (i.e, the degree of genuine differences between the studies and their results). Considering the 95% confidence interval, this positive effect of maternal position on the duration of labor may be merely marginal.

To critically appraise these results, the presence of any underlying potential sources of heterogeneity has to be examined. Typically, heterogeneity is associated with reporting bias, differences in: the intensity of interventions, the underlying risk, the effect size according to the study sample size and irregularities of data.

Analysis of Figures 2 to 6 may suggest the occurrence of a "small study effect". The small study effect is the trend for smaller studies in a meta-analysis to show larger treatment effects and it is also associated with reporting bias [25]. In this systematic review, two studies accounted for approximately 76% of the total reported sample (large studies) [10,14], two studies accounted for almost 14% of the sample (intermediate studies) [12,15], while five studies, each with fewer than 100 participants, accounted for approximately 10% of the total reported sample (small studies) [2,8,9,11,13]. Using the study performed by Flynn et al [2] as an example of the small study effect, this study accounts for approximately 3% of the total meta-analysis sample, but may have a much greater weight in the analysis (Figure 2). There is some controversy regarding how to deal with the small study effect. Simulation of exclusion of the Flynn study would change the conclusion of this meta-analysis (duration of first stage of labor, WMD (random) = -0.65; 95%CI -1.43-0.13).

Another possible source of heterogeneity in the present meta-analysis is the intensity of intervention in the studies included and the occurrence of several degrees of contamination (provision of the intervention to the control group) and co-intervention (provision of unintended additional care to either comparison group), as noted in Table 1. On the other hand, the occurrence of a performance bias in one of the larger studies was observed [10]. In this study, amniotomy was performed later in the study group. Considering that early amniotomy is associated with a reduction in the duration of first stage of labor [24], it is possible that the delay in performing amniotomy counterbalanced any possible effect of ambulation or standing in the upright position during the first stage of labor in that study.

The adoption of an upright position or walking during labor possible interferes on the performance of other interventions such as amniotomy, analgesia and monitoring during labor. The reverse way of this statement is also valid and this fact makes the isolation of possible effects of upright position or walking during labor a difficult task.

The evaluation of secondary outcomes suggests that the upright maternal position is a safe intervention. At the same time, while it produces no apparent benefit, neither does it appear to do any harm. The effect on maternal comfort is unclear, but freedom of movement may benefit patients individually.

## Conclusion

Adoption of the upright position or ambulation during first stage of labor may be safe, but considering the available evidence and its consistency, it cannot be recommended as an effective intervention to reduce duration of the first stage of labor.

Additional well-designed studies are warranted to further clarify the effectiveness of maternal position on the duration of labor and other outcomes.

## Competing interests

JPS declares that he has no competing interests. MAM, JGC, MYM are the authors of one of the studies included in the present systematic review.

## Authors' contributions

JPS and JGC participated in all the steps of the project, including project development, data extraction, data analysis and writing the final report. JGC, MAM and MYM took the initiative to develop the protocol for this systematic review. JPS and MAM were responsible for implementing the search strategy, the citation eligibility assessment and data extraction. JPS and JGC were responsible for the critical appraisal. All authors provided suggestions for the manuscript, read it carefully, agreed on its content and approved the final version.
